# Midfacial degloving approach for management of the maxillary fibrous dysplasia: a case report

**DOI:** 10.1186/s40902-018-0177-x

**Published:** 2018-12-06

**Authors:** Miju Kang, Yu-jin Jee, Deok won Lee, Sang-pil Jung, Se-won Kim, Sunin Yang, Dong-mok Ryu

**Affiliations:** 1 Department of Oral and Maxillofacial Surgery, Dental Hospital, Kyung-hee University Hospital at Gang-dong, #892 Dongnam-ro, Gangdong-gu, Seoul, 05278 Republic of Korea; 2 Department of Oral and Maxillofacial Surgery, College of Dentistry, School of Dentistry, Kyung-Hee University, 7-13, Kyungheedae-ro 6-gil, Dongdaemun-gu, Seoul, 02453 Republic of Korea

**Keywords:** Fibrous dysplasia, Midfacial degloving, Fibro-osseous lesion, Benign tumor

## Abstract

**Background:**

Fibrous dysplasia (FD) is a benign bone lesion characterized by the progressive replacement of normal bone with fibro-osseous connective tissue. The maxilla is the most commonly affected area of facial bone, resulting in facial asymmetry and functional disorders. Surgery is an effective management option and involves removing the diseased bone via an intraoral approach: conservative bone shaving or radical excision and reconstruction.

**Case presentation:**

This case report describes a monostotic fibrous dysplasia in which the patient’s right midface had a prominent appearance. The asymmetric maxillary area was surgically recontoured via the midfacial degloving approach under general anesthesia. Follow-up photography and radiographic imaging after surgery showed the structures were in a stable state without recurrence of the FD lesion. Furthermore, there were no visible scars or functional disability, and the patient reported no postoperative discomfort.

**Conclusions:**

In conclusion, the midfacial degloving approach for treatment of maxillary fibrous dysplasia is a reliable and successful treatment option. Without visible scars and virtually free of postoperative functional disability, this approach offers good exposure of the middle third of the face for treatment of maxillary fibrous dysplasia with excellent cosmetic outcomes.

## Background

Fibrous dysplasia (FD) is a benign but chronic fibro-osseous lesion that frequently occurs in the craniofacial region [1]. The precise origin of this disease is not clearly understood, but it is well known that normal bone is gradually replaced by an abnormal proliferation of fibro-osseous connective tissue. A possible mechanism may be the polyzygotic activated mutation of GNAS1 gene, encoding the a-subunit of the Gs stimulatory protein, localized at 20q13 chromosome within somatic cells during embryogenesis [2, 3]. FD involving the facial bones is often found as a slow-growing asymptomatic facial mass in young children which stops growing in the late teens or early twenties [4, 5]. The maxilla is the most commonly affected facial bone, with facial asymmetry and functional disorders being the usual complaints [1, 3].

Monostotic fibrous dysplasia, characterized by the involvement of only one bone with no systemic manifestations, is the most common form of this disease. The polyostotic form is more extensive, involves multiple regions, and may be associated with abnormal skin pigmentation, premature sexual development, and hyperthyroidism; this clinical constellation is known as McCune–Albright syndrome [1, 3]. Malignant degeneration occurs in approximately 0.5% of all patients, but more commonly arises in patients with the polyostotic form. Malignant changes may be associated with rapid tumor growth, pain, intralesional necrosis, bleeding, and elevation of the serum alkaline phosphatase level [6, 7].

Surgery is an effective management option for FD patients. Treatment of FD can vary and depends on the patient’s age and the aggressiveness, extent, and site of the lesion [8, 9]. If FD results in severe deformity or physiological dysfunction, total resection and reconstruction with a bone graft is the ideal procedure. However, mild disfigurement can be managed by simple shaving or other alternatives [9–11].

Until now, cranial fibrous dysplasia was accessed using a buccogingival or bicoronal approach. However, lesion resection is often incomplete when using the conventional, unilateral, buccogingival approach due to limitations in visualizing the lesion. This report describes a case of monostotic maxillary fibrous dysplasia with mild right facial deformity in which we adapted the midfacial degloving approach, a method that has been primarily used for paranasal sinus lesions or nasopharyngeal tumors [12, 13].

## Case presentation

**Fig. 1:**
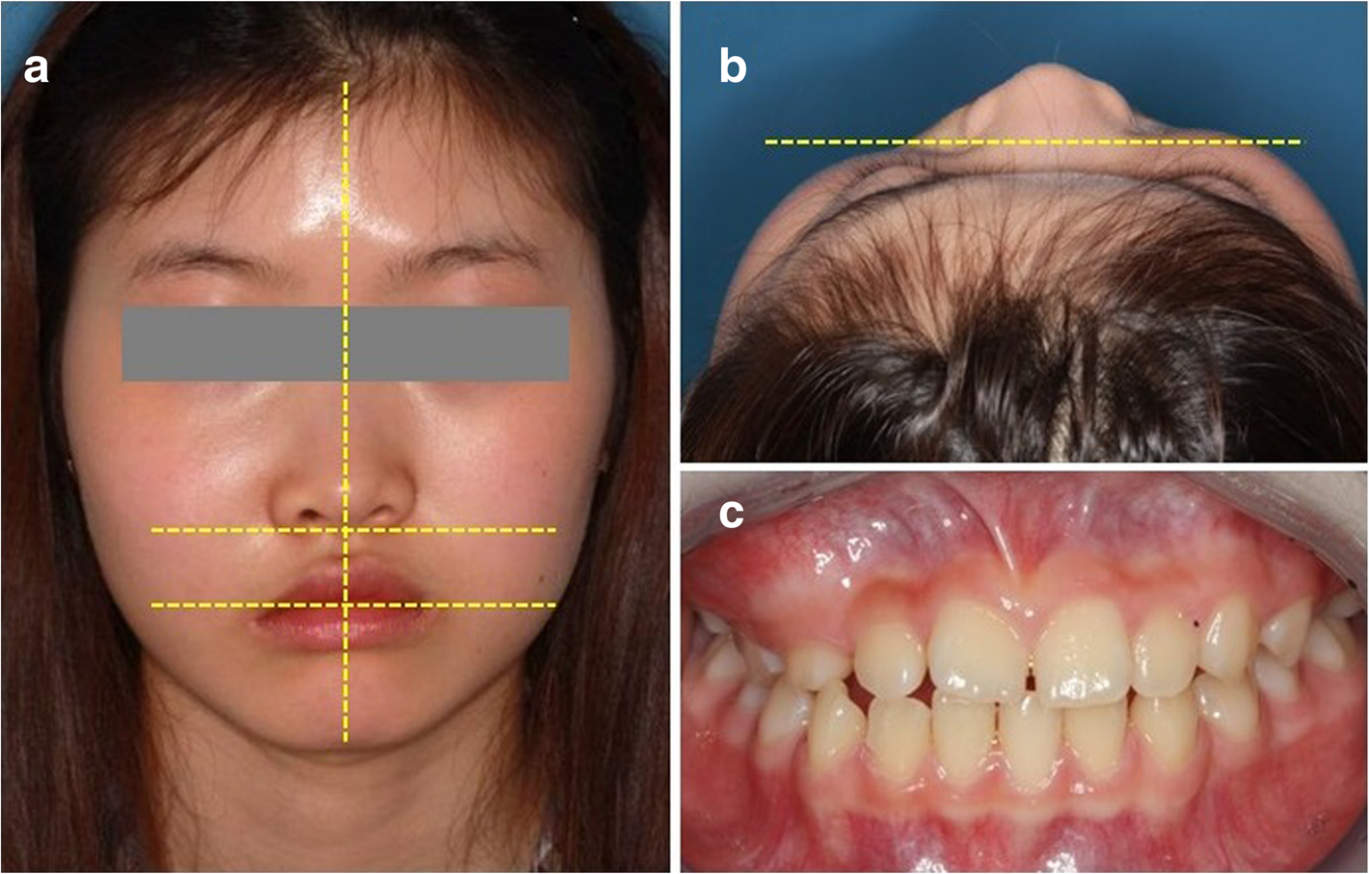
Preoperative view of enlargement of the right midface. **a**, **b** Preoperative photographs showed the enlargement of right face. The ala base and mouth corner on the right side were depressed by the fibrous dysplasia lesion. **c** Intraoral photograph showed gingival swelling on the right posterior area

An 11-year-old girl was referred to our hospital in August 2010 with a lesion in the right cheek area which was progressively enlarging. The patient complained that her nose and mouth corner were crooked and that her face was swelling. In clinical examination, the patient had a slight asymmetry in the right midface as a result of buccal and palatal cortical expansion from the right maxillary canine to the molar region, resulting in depression of the nasal alar and mouth corner (Fig. 1).

**Fig. 2:**
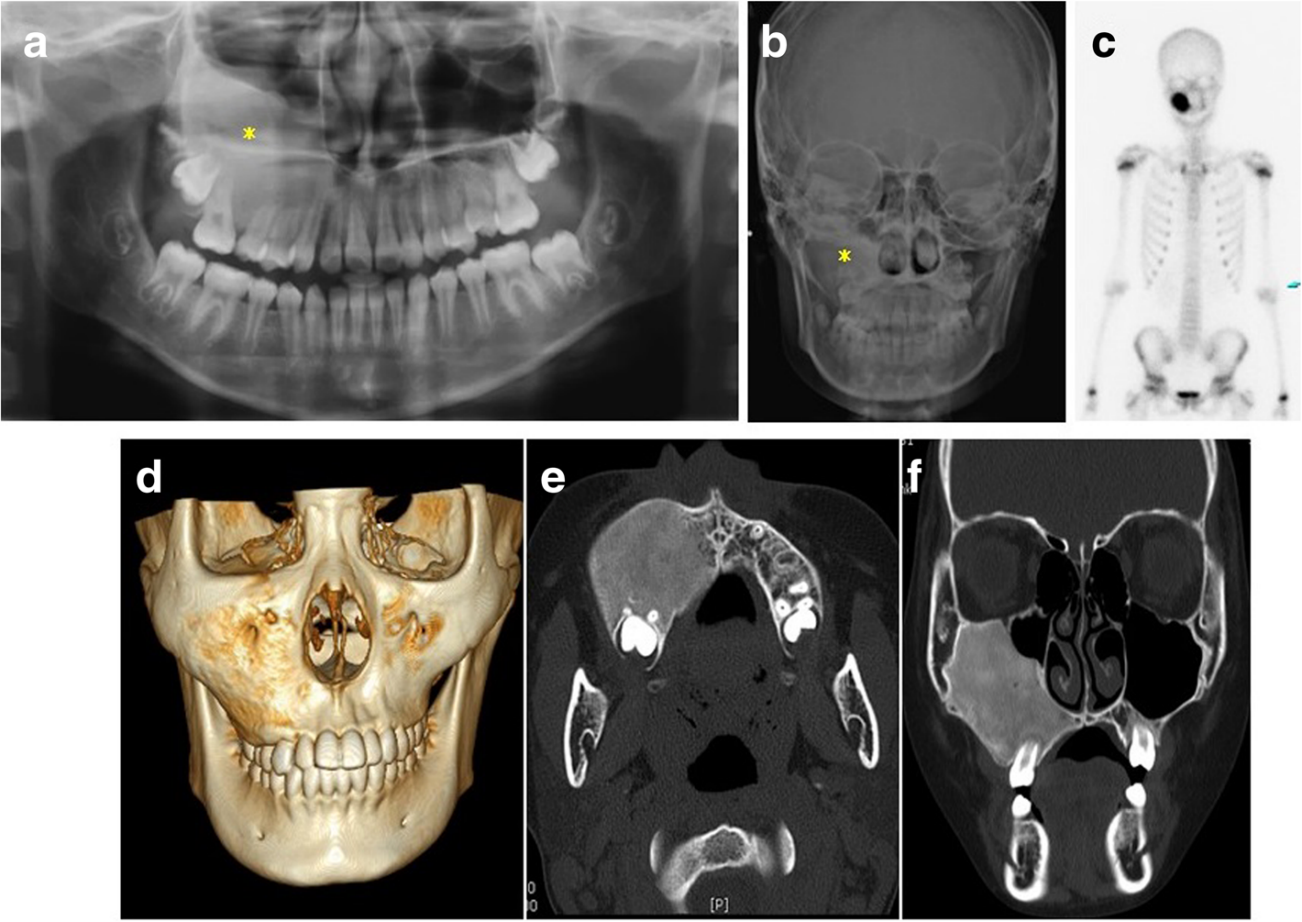
Preoperative radiographic images. **a** Panoramic view showed a high bone density on the right maxilla and zygoma and obliteration of the right maxillary sinus (asterisk indicates the fibrous dysplasia lesions). Additionally, the development of tooth germ was observed in the lesion. **b** Posteroanterior (PA) cephalometric view also showed an increased bone density on the right maxillary area and an asymmetric facial contour. **c** Bone scan showed an irregularly shaped hot uptake in the right maxilla, and no abnormally increased uptake was observed in any other sites. **d** Preoperative 3D CT image showed the enlarged right maxilla and zygomatic body. **e**, **f** Computed tomography (CT) showed 4.5 × 4 × 4.5 cm expansile ground-glass opacity lesion widely involving the right maxillary sinus, right maxillary alveolar process, and hard palate

A panoramic radiograph showed an increased bone density on the right maxilla and zygoma and obliteration of the right maxillary sinus. Computed tomography (CT) revealed a 4.5 × 4 × 4.5 cm, expansile ground-glass opacity lesion involving the right maxillary sinus, right maxillary alveolar process, zygoma, and hard palate. Bone scan revealed an irregularly shaped hot uptake in the right maxilla, and no abnormally increased uptake was observed at any other sites (Fig. 2). The physical examination did not show any other lesions, and the patient had no history of pain, trauma, loosening of teeth, or trismus. Based on the typical radiologic findings, the patient was diagnosed with FD, and no additional biopsy was performed. The patient had regular follow-up every 6 months to monitor the lesion’s progress. At the 1-year follow-up, the development of tooth germ within the lesion was normal, and slight expansion of the lesion to the bucco-lingual side was observed. Because we thought the patient was still growing and increasing in height, we decided to conduct an ongoing progress observation.

**Fig. 3:**
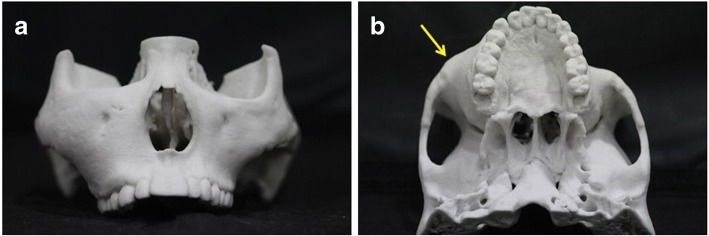
Preoperative skull model. **a**, **b** Arrows indicate the location of the midfacial fibrous dysplasia lesion. The right maxilla and zygomatic body were enlarged with an asymmetric shape

About 3 years later in December 2012, there were no significant changes of the FD lesion, but the distance from the mouth corner to the inner canthus was about 2 cm longer on the right side than on the left. Periodic observation was continued, and in August 2017, corrective surgery was planned because the maturation of the lesion was confirmed to be complete and there were no changes in the size of the lesion. At that time, the distance from the mouth corner to the inner canthus was 2.5 cm longer on the right side than on the left, and the distance from the occlusal plane to the outer canthus was 5 cm longer on the right side than on the left. Bone contouring surgery, the primary treatment for facial asymmetry and fibrotic bone lesions, was planned (Fig. 3).

The patient wanted to improve asymmetrical facial appearance through the surgery. Therefore, we aimed not only to remove the FD lesion, but also to make the patient’s facial as symmetrical as possible. For this, direct visualization and surgical approach to the infraorbital rim and lateral area of zygoma were required, but the surgical approach through buccogingival incision had limited access to these areas. On the other hand, the midfacial degloving approach was expected to help reestablishing symmetric facial contour by allowing direct comparison of the lesion with the normal side. Moreover, this approach provides esthetically acceptable outcomes, leaving no scars and no functional disability. Therefore, we decided to perform the operation through the midfacial degloving approach.

**Fig. 4:**
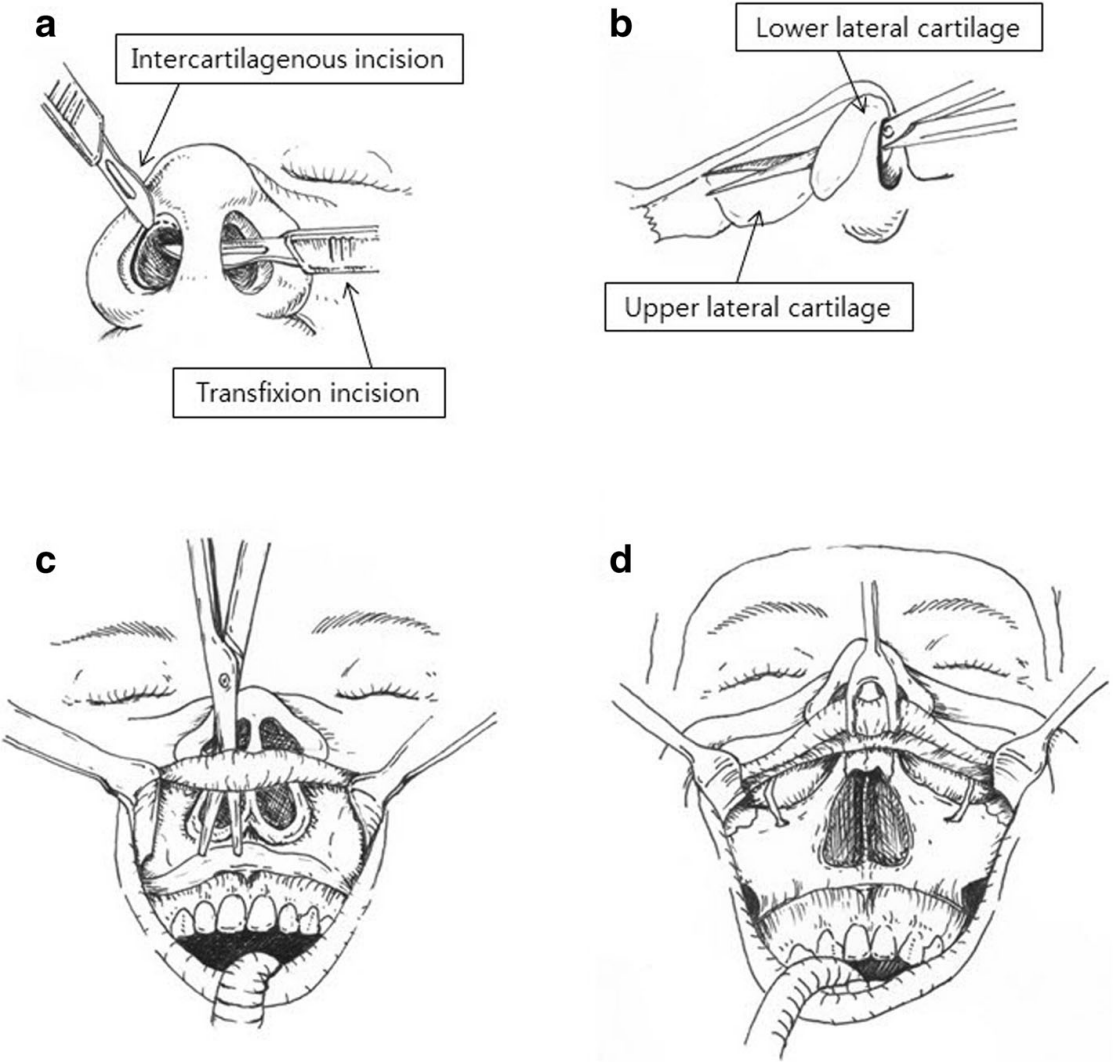
Schema of the midfacial degloving approach. **a** The intercartilaginous incision (between the upper and lower lateral cartilage) and the transfixion incision (anterior of the nasal septum). **b** Undermining with the scissor at the outer surface of the upper lateral cartilage. **c** Connection of the intranasal incisions and the oral incision. **d** Facial degloving to expose entire midface skeleton

With the patient under orotracheal anesthesia, the lesion was removed by the midfacial degloving surgical procedure. Local anesthesia with 2% lidocaine with epinephrine (1:100,000) was infiltrated into the maxillary vestibular mucosa and into the nose. The procedure is performed with a maxillary vestibular incision and three intranasal incisions to expose the entire midface skeleton that include (1) bilateral intercartilaginous, (2) complete transfixion, and (3) bilateral piriform aperture incisions (Fig. 4).

A buccogingival incision was made in the maxillary vestibule approximately 5 mm superior to the mucogingival junction and extended from the second molar to the contralateral second molar. Periosteal elevators were used to elevate the tissues in the subperiosteal plane fist over the anterior maxilla and then extending widely to encompass posterior tissues behind the zygomaticomaxillary buttress. The infraorbital neurovascular bundle was identified superiorly and dissected. Subperiosteal dissection along the piriform aperture stripped the attachments of the nasal labial muscularture to allow its complete release from the midface skeleton. The mucoperiosteal flap was elevated up to the piriform aperture.

The intercartilaginous incision divided the junction between the upper and lower lateral cartilages (Fig. 4b). An incision was made along the inferior border of the upper lateral cartilage, beginning at the lateral end and extending medially curved into the membranous septum anterior to meet transfixion incision (Fig. 4a). Laterally, the incision was sufficient that it extended to the piriform aperture. The lower lateral cartilage was eventually displaced superiorly during the degloving procedure, whereas the upper lateral cartilage remained attached to the midface skeleton. The transfixion incision was used to separate the membrane septum/columella from the cartilaginous septum. An incision was made along the caudal border of the septal cartilage from the medial end of the intercartilaginous incision toward the anterior spine (Fig. 4a). The intranasal incision was made by a full-thickness incision down through the periosteum of the piriform margin and the nasal floor.

Dissection through the intercarilaginous incision allowed access to the nasal dorsum and bones (Fig. 4b). Sharp subperichondrial dissection with a scalpel or a blunt dissection with scissors freed the soft tissues above the upper lateral cartilage as in a standard open rhinoplasty. The dissection should be within the subperichondrium plane to prevent injury to the overlying musculature and blood vessels of the nose. Elevation extended laterally to the nasomaxillary sutures and superiorly to the glabella. Retraction of the freed soft tissues allowed sharp incision to be made with a scalpel or with sharp periosteal elevators through the periosteum at the inferior edge of the nasal bones. Elevation of the soft tissue laterally to the piriform aperture was also performed so that the maxillary vestibular dissection was easily connected to this pocket.

**Fig. 5:**
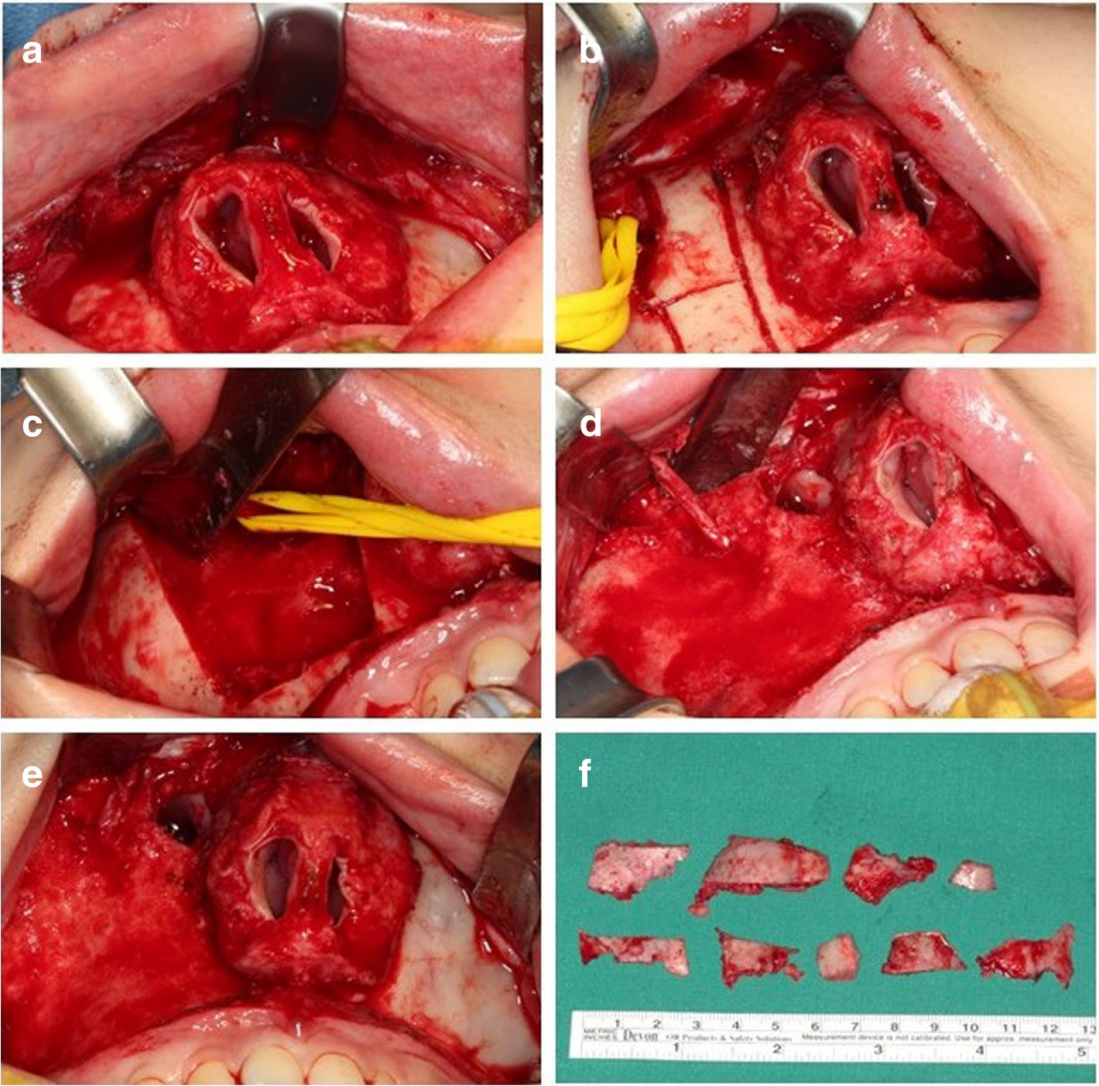
Intraoperative views of the midfacial degloving approach. **a**, **b** Intraoperative view of the midfacial degloving technique showing exposure of the nasal cavity and maxillary area. **c**, **d** The bilateral maxillary areas were completely exposed, and the bilateral fibrocystic lesion was removed while preserving the infraorbital nerve. **e**, **f** The right maxillary area from which the lesion was removed and the left area were almost symmetric

After the connection of the nasal and oral incisions, the midface was degloved. The midface skin was separated from the maxilla and the nasal pyramid. The upper lip and the intact nasal columella, nasal tip, and alar cartilages were then retracted by two Penrose drains introduced through the nostrils over the nose to the level of the inferior orbital rim. This approach provided visualization of the medial maxillary wall, pterygoid junction, nasofrontal suture, infraorbital rim, and laterally to the temporal process of the zygoma (Fig. 4d). Under direct visualization, the overgrowing bone lesion was then excised using osteotomes and saws. The right maxilla was drilled further at the orbital rim and laterally till zygomatic complex. The contour of the midface was reestablished using burr to give a cosmetically normal looking midfacial skeletal contour while protecting the infraorbital nerve (Fig. 5). For the removed lesion, a biopsy was performed for the accurate diagnosis and histologically confirmed as FD. The soft tissues were then carefully redraped and the nasal tip brought back into position. The intranasal incisions were closed using 4-0 resorbable sutures (vicryl), and the transfixion sutures were precisely performed to determine the final position of the nasal tip and prevent vestibular stenosis. The cinch suture of alar base was used to prevent postoperative alar base widening. The intraoral incisions were closed using a 3-0 black silk. Nasal packing into the maxillary dead space with Vaseline gauze was done for 3 days in order to minimize the postoperative bleedings.

**Fig. 6:**
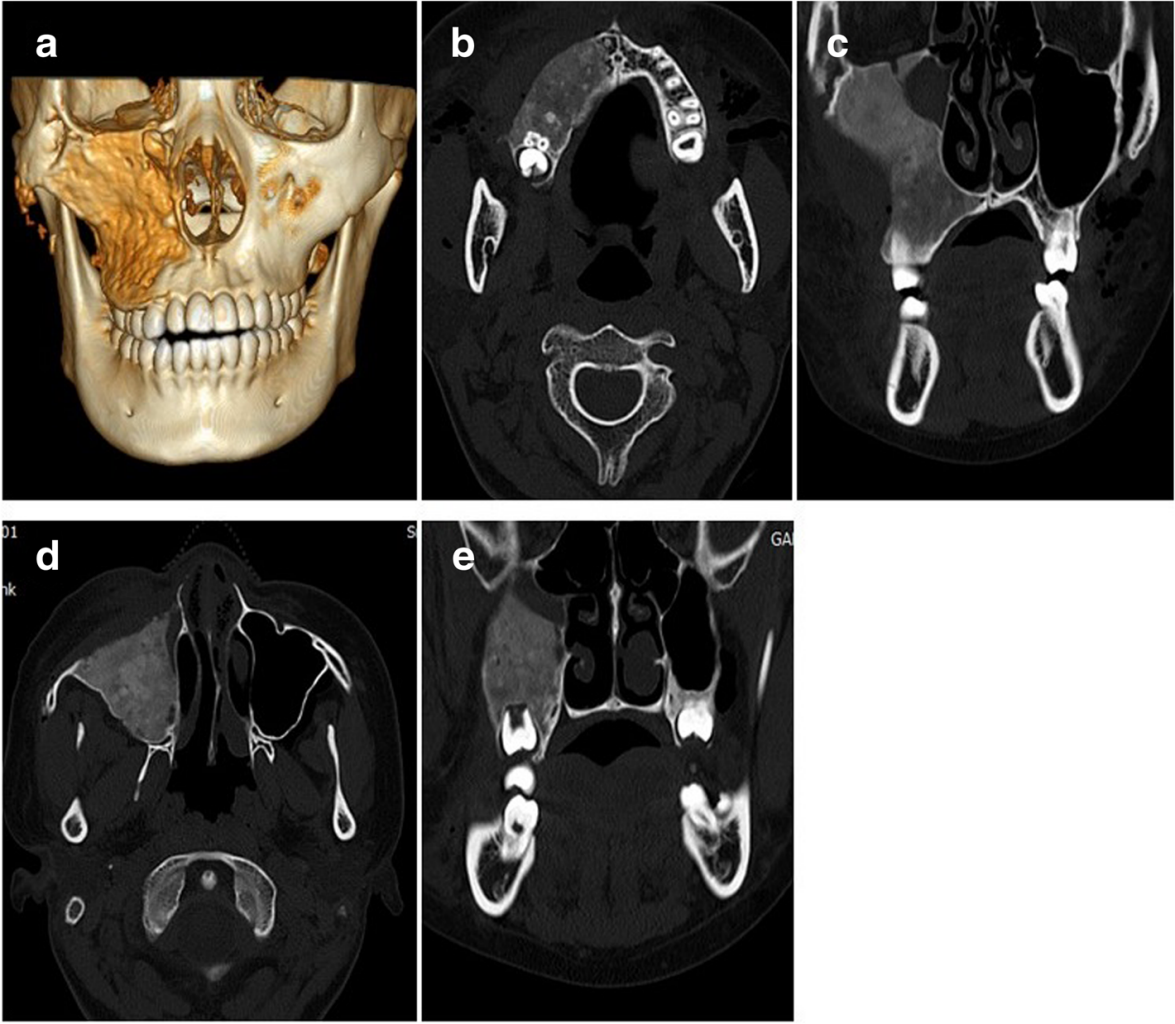
Postoperative radiographic images. **a** Postoperative 3D CT image showed relatively symmetrical skeletal contours and cortical bone formation on surgical site. **b**, **d** Four-month postoperative axial CT showed the restoration of the anterior protrusion of the maxilla. **c**, **e** Four-month postoperative coronal CT showed the improved contour of the right maxilla

**Fig. 7:**
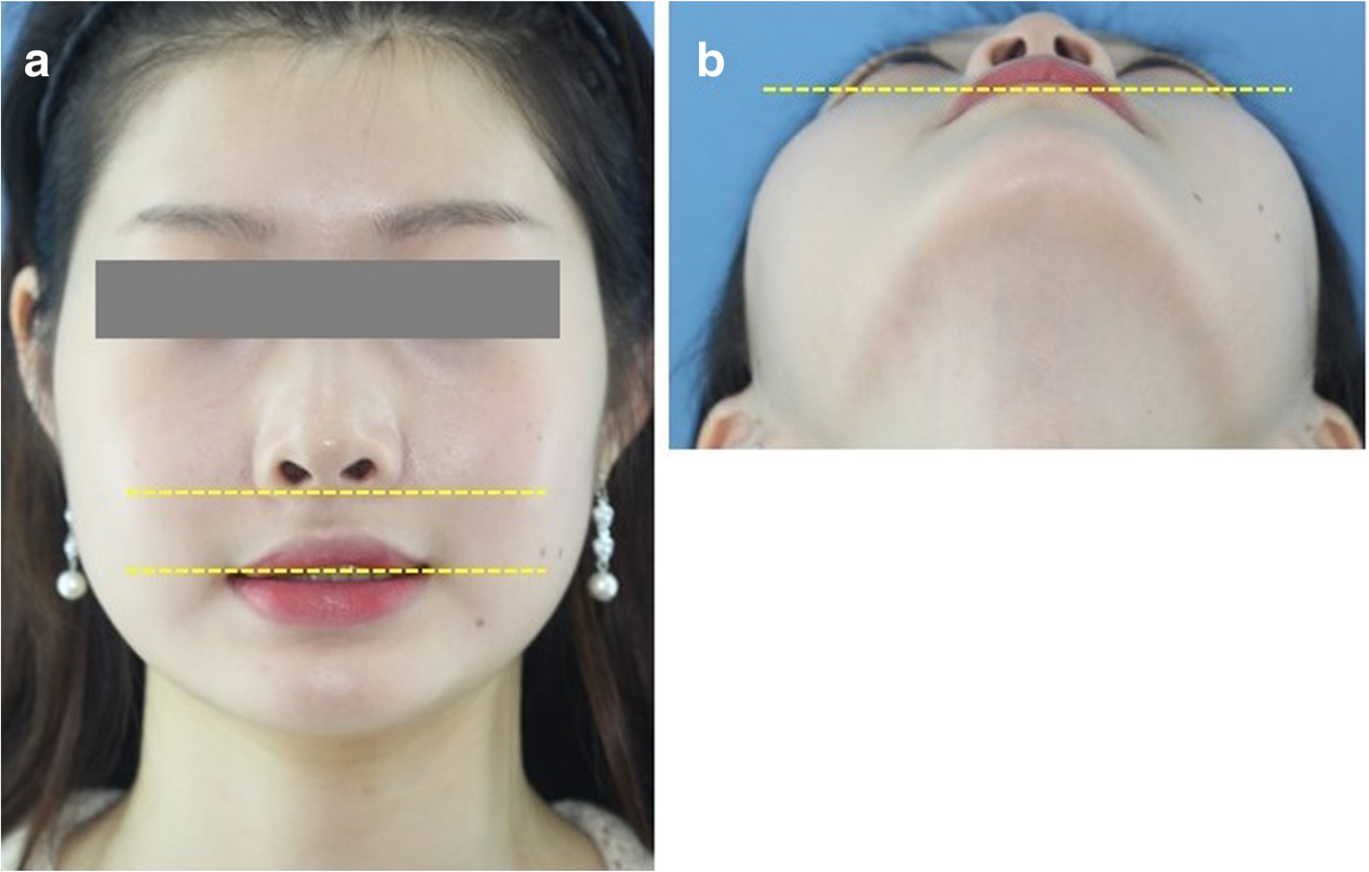
Appearance at 1 year’s follow-up showing symmetry of the face. **a**, **b** Postoperative photographs showed that the deviation of the ala base and mouth corner was almost resolved. Note the improved facial symmetry and contour of the right maxillary alveolus

The patient’s postoperative course was generally uneventful. There was moderate nasal crusting for 3–4 days. Mild swelling with periorbital ecchymosis disappeared after 2 weeks, and transient paresthesia around the infraorbital nerve spontaneously resolved after 3 month. No postoperative complications such as epistaxis, vestibular stenosis, or esthetic problems of the nose were seen. Clinical and radiographic examinations obtained 4 months after surgery showed the anatomical structures were in a stable state without recurrence of FD (Fig. 6). The esthetic result was satisfactory for the patient, and occlusal state was also well maintained (Fig. 7). Therefore, no additional orthodontic treatment or orthognathic surgery was performed.

## Discussion

Fibrous dysplasia (FD) is a slowly progressing bone lesion resulting from the displacement of normal medullary bone with abnormal fibro-osseous connective tissue. FD can be classified as monostotic when it involves a single bone and polyostotic when it involves multiple bones [1]. The former has an incidence of 70–80%, while the latter may be seen in 20–30% of patients with FD [13–15]. The monostotic form is usually asymptomatic, whereas the polyostotic form, accompanied by various endocrine disorders, irregular skin pigmentation, and early sexual maturity, is called McCune–Albright syndrome [1, 3, 16]. The monostotic type is more prevalent in the maxilla in the craniomaxillofacial skeleton [1, 3, 17]. In a patient whose only clinical feature is monostotic fibrous dysplasia, biopsy and identification of a somatic gene mutation may be required to confirm the diagnosis [1]. However, many experienced clinicians and radiologists will often notice that a lesion appears characteristic of FD on radiographic examination because it is an expansile and intramedullary lesion with a ground-glass appearance, thus diagnosing FD without the need for biopsy in typical cases [3]. Diagnosis can also be made by histologic examination of the biopsied tissue. The typical microscopic features of FD include a background of loosely arranged fibrous stroma with irregularly shaped bony trabeculae. The bone trabeculae are thin and disconnected to each other and have been described as “Chinese characters” [1, 3, 12, 18].

The differential diagnosis of the condition includes ossifying fibromas, cemento-ossifying fibroma, cemento-osseous dysplasias, aneurysmal bone cyst, cherubism, and giant-cell granuloma [1, 3, 19]. The lesions are grouped together because of their common histological features that include fibrous material mixed with bony structures and some elements of irregular woven bone [1, 12].

FD in the craniofacial area can cause significant expansion of the bones with facial asymmetry and disfigurement. In some cases, FD can cause displacement of structures, such as the orbit that can lead to visual disturbances and the auditory canals that can lead to hearing impairment [20]. The progression of the lesion into the oral cavity can compromise masticating and speaking. Periodontal and occlusal changes may also present, and this condition may require orthodontic treatment and orthognathic surgery to correct the malposition of the involved teeth and jaw [21].

Many clinical studies have reported that the appropriate treatment for this lesion depends on its location, its effect on function, and, ultimately, cosmetics. Oftentimes, the monostotic form is incidentally discovered on radiographs, and it is usually asymptomatic. Such lesions ordinarily pose no risk for pathologic fracture or deformity, and only clinical observation is warranted. Follow-up radiographs should be made every 6 months to verify that there has been no progression [13].

Surgical therapy is an effective management option for patients with cosmetic problems and deformities, and it is the preferred treatment to avoid pathological fractures and remove symptomatic lesions [9–11]. The available treatment options include radical resection, conservative contouring, and curettage. Conservative shaving or contouring has been recommended by some authors [18, 22] who maintain that periodic contouring could be performed until a static phase is reached. Total resection and reconstruction with a bone graft is considered to be the standard surgical procedure in cases of moderate to severe fibrous dysplasia [7, 8]. However, because of the neurovascular structures and external incision scar, surgical intervention is often challenging.

Until now, some surgical accesses have been proposed for the treatment of FD in the midface, such as the buccogingival or bicoronal approach, the Weber–Ferguson incision, and the midfacial degloving approach [22, 23]. The selected approach should provide adequate exposure of the lesion, minimal scarring, and minimal risk of injury to nerves or other vital structures. However, midfacial FD tends to be under-corrected due to the limits of the approaching methods, even using the bicoronal approach. The Weber–Ferguson incision causes a large range of facial scars because of the external incisions. The buccogingival incision has limitations on direct access to the orbital rim and temporal process of zygoma. However, the midfacial degloving approach fulfills most of the above requirements. The midface degloving approach first described by Casson et al. [24] is useful in corrective surgery for broad midfacial deformities, as in our case. Although this procedure requires precise surgical technique and a thorough understanding of the anatomy of the midface, the advantage of this technique is excellent bilateral exposure of the mid third of the face, including the maxilla, paranasal sinus, and nasal cavity, without skin incision [25]. With this wide surgical field, we could perform a delicate osteotomy, shaving the projected portion of the facial bones under direct vision.

Indications for this procedure include a wide variety of benign maxillary or sinonasal conditions, such as ameloblastoma, inverted papilloma, and various odontogenic or nonodontogenic cysts [25]. Particularly advantageous is the use of this procedure in the management of locally aggressive, histologically benign lesions such as odontogenic keratocyst and ameloblastoma where complete removal of the lesion is recommended without facial incisions. This approach can also be used in benign fibro-osseous conditions, facial fractures, orthognathic surgery, and bone grafting [25, 26].

The most common complications of the midfacial degloving technique are moderate transnasal crusting and facial paresthesia [27, 28]. Nasal crusting is unavoidable as an immediate postoperative sequela but subsides as regrowth of mucosa occurs. Infraorbital numbness and paresthesia should resolve in few months if the infraorbital nerves were carefully preserved during surgery. The rate of permanent infraorbital anesthesia has been reported to be less than 3% [29]. Nasal cosmetic deformities are rare complications and can be avoided with precise intranasal reapproximation. Problems such as postoperative nasal bleeding, vestibular stenosis, and oronasal or oroantral fistula are infrequently encountered. Vestibular stenosis and fistula formation can be avoided if the incisions are sutured promptly and meticulously [29, 30]. Other complications may arise secondary to midface osteotomies, such as epiphora during a medial maxillectomy.

In the present case, a FD lesion in the right maxilla and zygoma area was successfully removed by the midfacial degloving approach. This technique ensured complete visualization of the zygomatic prominence and the infraorbital rim, which was helpful in identifying and controlling the symmetry and width of the face. Furthermore, wider exposure of the facial skeleton helped us to better estimate the normal facial contour. Despite excessive undermining of the midfacial region, our patient showed good cosmetic results without sagging of tissue, deviation of the nose, or flattened ala. Mild swelling and paresthesia around the infraorbital nerve resolved spontaneously, and occlusion state was well maintained.

Although the use of the midfacial degloving approach was rare in the treatment of FD, some published papers have reported very similar cases [31–33]. In patients in their teens and twenties, facial asymmetry and swelling were found, and no other symptoms such as pain or visual disturbances, loosening of teeth, or paraesthesia over the face were observed. Except for one case, other cases were unilateral maxillary FD. The range of the expansile lesions included the maxillary bone involving maxillary alveolar process, hard palate, zygoma, and medial wall of orbit. Since the typical buccogingival incision was limited to access to the lesions, surgery was performed using the midfacial degloving approach. The surgical procedures and methods that contour the facial bone were similar to this case. There were no recurrence of the lesion and serious complications, and the esthetic result was satisfactory for the patients. However, in one case that has bimaxillary and mandibular FD, allocartilage and orbital surgery for correction of severely deviated nose and severe orbital dystopia were required [33].

## Conclusion

Our case indicates that the midfacial degloving approach is a very effective and safe technique for the management of maxillary FD. We found that the midfacial degloving approach showed excellent exposure, a good symmetry outcome, no scars, and no cosmetic defects. These results indicate that this approach is superior to conventional buccogingival surgical approaches, especially in cases of extensive midfacial fibrous dysplasia.

## Abbreviations

CT: Computed tomography
FD: Fibrous dysplasia
